# An Examination of Personalized Approaches in the Management of Ankle Fractures: A Thorough Evaluation of Soft Tissue Factors, Treatment Methods, and Patient Adherence

**DOI:** 10.7759/cureus.45507

**Published:** 2023-09-18

**Authors:** Adrian Cursaru, Mihnea Popa, Alexandru Lupu, Sergiu Iordache, Mihai Costache, Bogdan Cretu, Bogdan Serban, Catalin Cirstoiu

**Affiliations:** 1 Orthopedics and Traumatology, Carol Davila University of Medicine and Pharmacy, Bucharest, ROU; 2 Orthopedics and Traumatology, University Emergency Hospital, Bucharest, ROU

**Keywords:** ankle trauma, minimally invasive intervention, treatment compliance, open fractures, surgical management, soft tissue damage, tibial pillar fractures

## Abstract

This study offers a thorough analysis of tibial pilon fractures, accounting for patient compliance, diverse treatment options, and soft tissue implications. The article discusses varied treatment pathways, ranging from single­stage interventions to two-stage methods for open fractures by presenting seven clinical cases. The emphasis is on the intricate interplay of trauma intensity, bone damage, and adjacent soft tissue in dictating treatment plans and patient outcomes. The challenges posed by non-compliant patients rejecting advised treatments are underscored, illuminating the inherent risks. Drawing from varied patient demographics, comorbidities, and fracture types, a comprehensive guide for clinicians emerges. The findings underscore the importance of a tailored, patient-centric approach, considering the multifaceted nature of ankle fractures, local soft tissue health, patient's overall well-being, and their adherence to the proposed treatment regimen.

## Introduction

Tibial pilon fractures, also known as distal tibial joint fractures, are a complex area of traumatology. These injuries occur in 3-10% of tibial fractures and less than 1% of lower extremity fractures. The accompanying fracture of the fibula is common, occurring in approximately 75-90% of cases. These fractures often result from high-energy traumas like falls from great heights or vehicular accidents, which exert heavy axial force and cause the tibial plafond to burst over the talus [1,2].

In 1911, Etienne Destot coined the term "Pilon" to metaphorically describe the crushing force of the distal tibia on the talus. Pilon fractures can also result from low-energy rotational forces, such as those in skiing accidents [3]. The surrounding soft tissue is often severely damaged by these fractures, and 6% of patients require intensive care due to multiple injuries. The management of these fractures is complicated by significant soft tissue injury and displaced articular fragments [4].

The wide range of injuries and patient factors, such as concomitant injuries, complicate the treatment of pilon fractures. Open reduction and internal fixation (ORIF) are common. However, due to widespread tissue edema and extensive soft tissue dissection, these can cause postoperative wound healing problems and infection, especially in the early phase of the fracture. To reduce complications and optimize fracture healing, external fixation followed by ORIF is used [3,5]. To reduce soft tissue injury, new surgical techniques have been developed. Double incisions, anterolateral, and lateral approaches have been tried to improve exposure while protecting the superficial peroneal nerve (SPN) and other vital structures. In open pilon fractures, wound dehiscence and deep tissue infections must be carefully managed, making surgical timing even more complicated. Restoring articular congruency and epiphyseal-metaphyseal alignment is the goal of treating these fractures. In addition to ORIF, limited internal fixation with an external fixator (LIFEF), intramedullary nailing, and minimally invasive plate osteosynthesis is increasingly used [6,7].

The multifactorial nature of tibial pilon fractures, the potential for soft tissue compromise, and the variety of treatment modalities demonstrate their complexity. Despite advances in surgical techniques and approaches, pilon fractures remain a diagnostic and therapeutic problem. This intricate interplay of forces, anatomy, surgical innovation, and individual patient factors makes the study of pilon fractures an essential part of modern orthopedics and a vibrant field of ongoing research and evolution [8,9].

## Materials and methods

The present study employed a retrospective analysis approach to investigate complex tibial pilon fractures that were treated at the Orthopedics and Traumatology Clinic of the Bucharest University Emergency Hospital. The temporal scope of the analysis spanned from July 2022 to July 2023.

Patient selection

For the purpose of this investigation, a cohort of seven complex and representative clinical cases was chosen to be included in our study. The surgical interventions performed on these cases were conducted by experienced senior surgeons from our facility who possess significant expertise in the fields of orthopedics and traumatology. The provided criteria function to both include and exclude specific cases, thereby facilitating a concentrated and authoritative examination of intricate scenarios within these specialized domains.

Surgical procedure

The patients were positioned in a supine decubitus posture on the operating table during the surgical procedures. The administration of either general anesthesia or lumbar anesthesia was determined based on the patient's preference and the complexity of the case. In order to achieve an ideal alignment, a strategically positioned elevation was placed under the hip on the same side to correct the outward rotation of the lower extremity and stabilize the positioning of the ankle.

Data collection

A thorough examination was performed on patients' records, encompassing radiological data, operative notes, and outpatient records. This facilitated a comprehensive comprehension of the interventions administered and their corresponding results.

Exclusion criteria were implemented in the study to ensure that only the most relevant cases were included, thereby maintaining a rigorous selection process. The study excluded individuals with stress fractures, pathological fractures, concomitant fractures in other limbs, segmental fractures, and a history of previous ankle surgeries.

Radiological evaluation

All participants included in the study underwent bilateral ankle radiographs in order to analyze the characteristics of the fractures and identify any associated underlying pathology. The evaluation of radiological imaging following surgery was consistently conducted to identify potential complications such as infections, nonunion, implant failure, and osteomyelitis.

Fracture classification

The fractures were systematically classified using the Rüedi/Allgöwer and Gustilo-Anderson classification systems. The utilization of this classification system facilitated the customization of the surgical approach and the anticipation of potential complications and outcomes.

Follow-up and outcomes

Subsequent to the surgical procedure, patients were subjected to postoperative monitoring in order to assess any potential complications and evaluate their recovery progress. The study focused on several key aspects, namely the effectiveness of surgical procedures, the impact of individual patient characteristics, associations with fracture categorizations, and the broader implications concerning tibial pilon fractures.

## Results

Particular focus is placed on cases exhibiting closed fractures coupled with good-quality soft tissues within the complex landscape of treating tibial pillar fractures. Through the anteromedial or anterolateral approach, these enable a single-stage surgical intervention that combines reduction and fixation. The protocol is especially effective for emergency room patients who arrive early, have little soft tissue edema, have no preoperative illnesses, and are not taking anticoagulants. These patients underwent prompt surgery within the first 24 hours. An anterolateral or anteromedial incision was used to gain access to the distal tibia while carefully protecting the tibialis anterior tendon. The extremity's length was restored after careful fracture line exposure and hematoma cleaning, and fragments were short-term fixed with K wires. The fractures were stabilized with either a 3.5 mm locked distal tibia anatomical plate or a 3.5 mm locked anteromedial pillar plate, supplemented with cannulated screws as necessary. Defective metaphyseal regions can be fortified with allografts if necessary. To ensure accuracy, fluoroscopy was used to confirm joint face restoration, plate orientation, and fracture fixation. After thorough saline cleaning, the wound was sutured without the aid of a drain.

Case one

**Figure 1:**
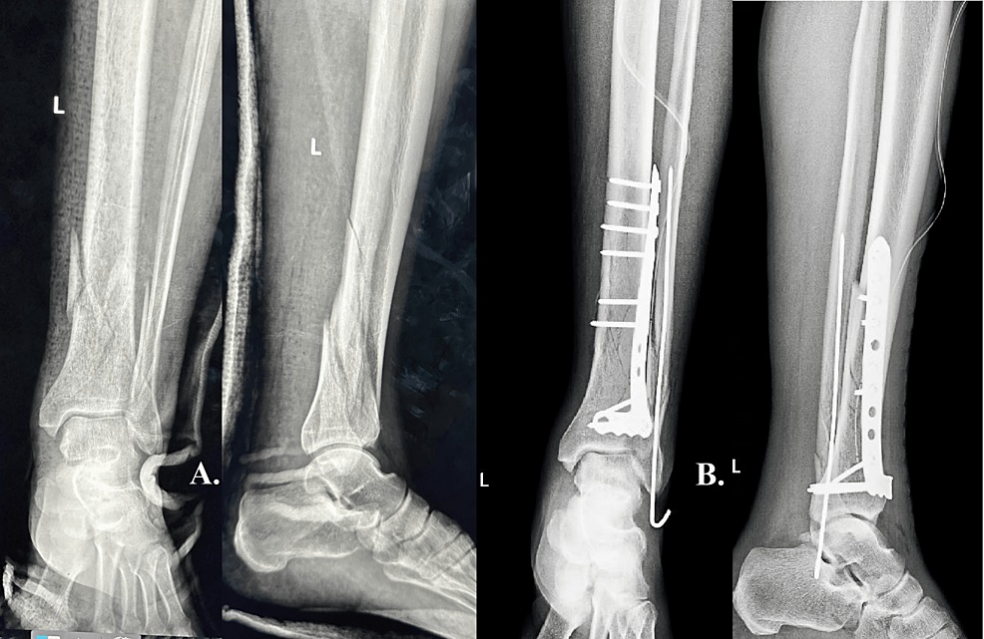
Case one A: shows a comminuted fracture involving the distal third of the tibia with concurrent extension into the tibial plafond (pillar) and a spiral fracture of the fibula. B: shows satisfactory intraoperative alignment and fixation.

For the first case, we present a comminuted fracture involving the distal third of the tibia with concurrent extension into the tibial plafond (pillar) and a spiral fracture of the fibula in a 42-year-old patient who had experienced acute trauma following a fall of about 2 meters (Figures 1A, 1B). The surgical strategy was guided by the radiological evidence and the clinical evaluation of soft tissue integrity. An open reduction and internal fixation (ORIF) procedure was chosen because there was no significant soft tissue compromise. This required fixing the fibula fracture with a Kirschner wire (K-wire) and placing an L-shaped plate at the site of the tibial fracture.

Case two

**Figure 2:**
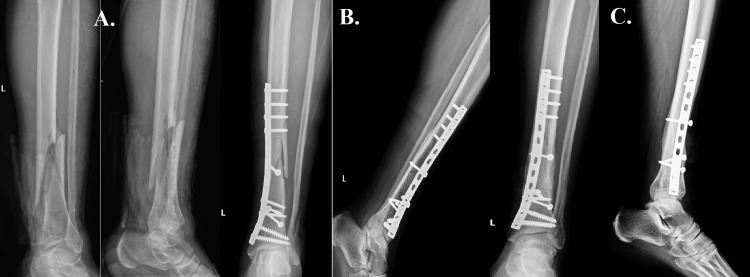
Case two A: shows a comminuted fracture affecting the lower third of the distal tibia after a traumatic injury while pivoting during a skiing accident. B: shows the alignment after the surgical reduction and fixation. C: follow-up radiographic imaging one year after surgery, shows a successful outcome with strong fracture consolidation, demonstrating the effectiveness of the surgical strategy and the patient's subsequent healing.

For the second case, we present a comminuted fracture affecting the lower third of the distal tibia in a 38-year-old patient who had suffered a traumatic injury while pivoting during a skiing accident (Figure 2A). Clinical examination showed that the patient was a prime candidate for immediate surgical intervention because the surrounding soft tissues were in good health. The treatment method of choice was open reduction and internal fixation (ORIF) (Figures 2B, 2C), with a straight medial plate being used to stabilize the fracture site.

Open fractures carry a higher risk of developing septic complications, with the risk varying depending on how severe the fracture is according to the Gustillo-Anderson classification. A primary reduction and fixation using a plate and screws may be considered in cases of Gustillo-Anderson I fractures. A two-stage surgical approach is required for more severe fractures. Within the first 24 hours, patients who have open fractures and severe soft tissue edema are treated right away with a tibio-calcaneal external fixation. The external fixator is secured with transmission screws that are inserted through the tibia and calcaneus under fluoroscopic guidance to achieve the best distraction and reduction of the fracture line. After clinical and laboratory monitoring, deep vein thrombosis (DVT) prophylaxis, and triple prophylactic antibiotic therapy, the patients are then discharged. The external fixator is aseptically removed once the edema has sufficiently subsided and the readiness is determined by the prick test. Then, similar to single-stage surgical procedures, open reduction and internal fixation (ORIF) is carried out. By carefully following a two-step procedure, the risk of infection and circulatory issues is reduced while proper fracture alignment and healing are guaranteed.

Case three

**Figure 3:**
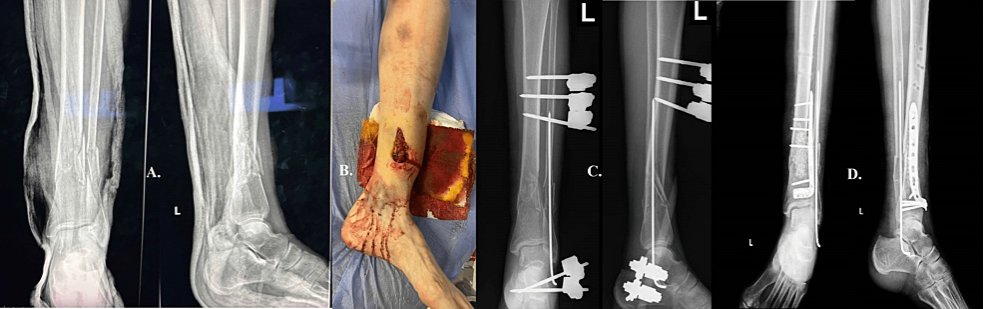
Case three A: shows the initial plaster cast immobilization. B: shows the 4 cm open wound on the inner side of the distal tibia communicating with the bone. C: an external fixator was used to stabilize the fracture. D: The second surgical intervention used a plate and screws for reduction and osteosynthesis.

A 54-year-old patient, having suffered a fall down the stairs, arrived in the emergency room displaying a classic Gustilo-Anderson (GA) IIIA open fracture, the fracture was accompanied by a 4 cm long open wound on the inner side of the distal tibia, which was visibly communicating with the bone (Figures 3A, 3B). The initial intervention encompassed prompt irrigation and immobilization through the application of a plaster cast, which was subsequently followed by surgical intervention. The surgical procedure involved a comprehensive cleansing and removal of necrotic tissue from the affected regions, followed by the application of an external fixator to stabilize the fracture (Figure 3C). The meticulousness exhibited during the initial treatment phase played a pivotal role in safeguarding the integrity of the fracture site. Following a three-week period after the occurrence of trauma, during which there was a positive local progression, a subsequent surgical intervention was performed. The procedure involved the utilization of a plate and screws for reduction and osteosynthesis, along with the strategic positioning of antibiotic-impregnated calcium pearls at the fracture site to minimize the potential development of secondary septic progression (Figure 3D).

In specific clinical contexts, particularly when managing patients with comorbidities or increased surgical risk, a minimally invasive intervention may be the preferred modality. Although this approach does not offer the same level of mechanical stability as conventional, more invasive methods, it is proficient in successfully repairing the fracture. By employing a variety of specialized instruments and employing targeted procedures, the surgeon is able to effectively align and stabilize the fracture fragments while minimizing any potential disruption to the surrounding tissues. The primary focus of this approach is to prioritize the patient's well-being, with an emphasis on attaining a favorable outcome that effectively balances the healing of fractures and the imperative to minimize surgical trauma. The aforementioned strategy highlights the significance of tailoring treatment planning to the specific requirements and situations of individual patients, thereby enabling the surgical approach to be guided accordingly.

Case four

**Figure 4:**
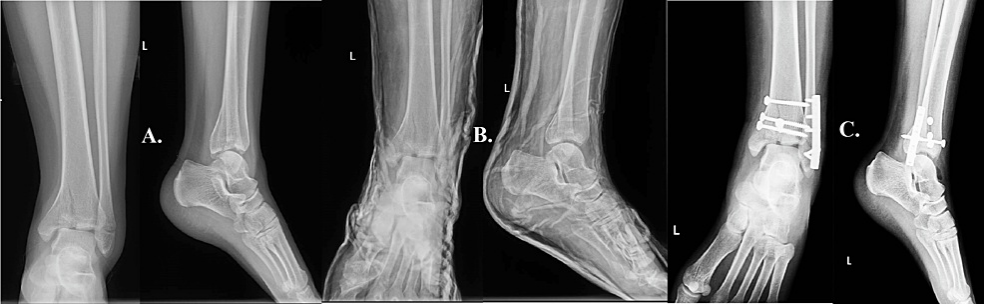
Case four A: low-energy ankle trauma caused the tibia and fibula intra-articular fracture. B: the patient's complex medical history led to the initial plaster cast immobilization of the injury. C: shows the conservative approach to fracture reduction and fixation, which minimized tissue damage and surgical stress on the delicate patient.

This case study examines the application of minimally invasive treatment in a 65-year-old patient with stage II renal failure, diabetes mellitus, a history of stroke, and angina pectoris. The administration of the treatment occurred subsequent to a low-energy ankle trauma. The provided radiography offers a concise depiction of the intra-articular fracture that transpires at the tibia and fibula level (Figure 4A). As a result of the patient's complex medical history, a cautious approach was adopted, leading to the initial implementation of a plaster cast for the purpose of immobilizing the injury (Figure 4B). Given the significant significance of mitigating surgical stress on patients with fragile medical conditions, the overseeing physician opted to adopt a conservative strategy for managing the fracture. The utilization of this methodology enabled successful reduction and stabilization of the fractures while mitigating harm to the surrounding tissue (Figure 4C).

Adherence to prescribed treatment is of paramount importance in the management of tibial pillar fractures, as it significantly influences the patient's recovery process. Nevertheless, there is a distinct and demanding group of patients who, despite being provided with thorough information and instructions by their healthcare provider, consciously choose not to adhere to the recommended treatment. The patient's initial refusal and subsequent delays in treatment not only heightened the intricacy of the intervention but may have also influenced the long-term prognosis. The act of refusing, which frequently arises from individual beliefs or misunderstandings, can significantly complicate the process of recovery. Non-adherence to prescribed medical protocols can give rise to avoidable hazards, such as delayed union, malunion, infection, or the emergence of chronic ailments, thereby potentially compromising the overall quality of life. The responsibilities of physicians extend beyond surgical procedures to include patient education and motivation, with the aim of promoting adherence to medical recommendations. However, instances in which patients continue to refuse treatment serve as a reminder to medical professionals of the intricate relationship between medical science and individual autonomy, and the resulting complexities that can emerge from this dynamic.

Case five

**Figure 5:**
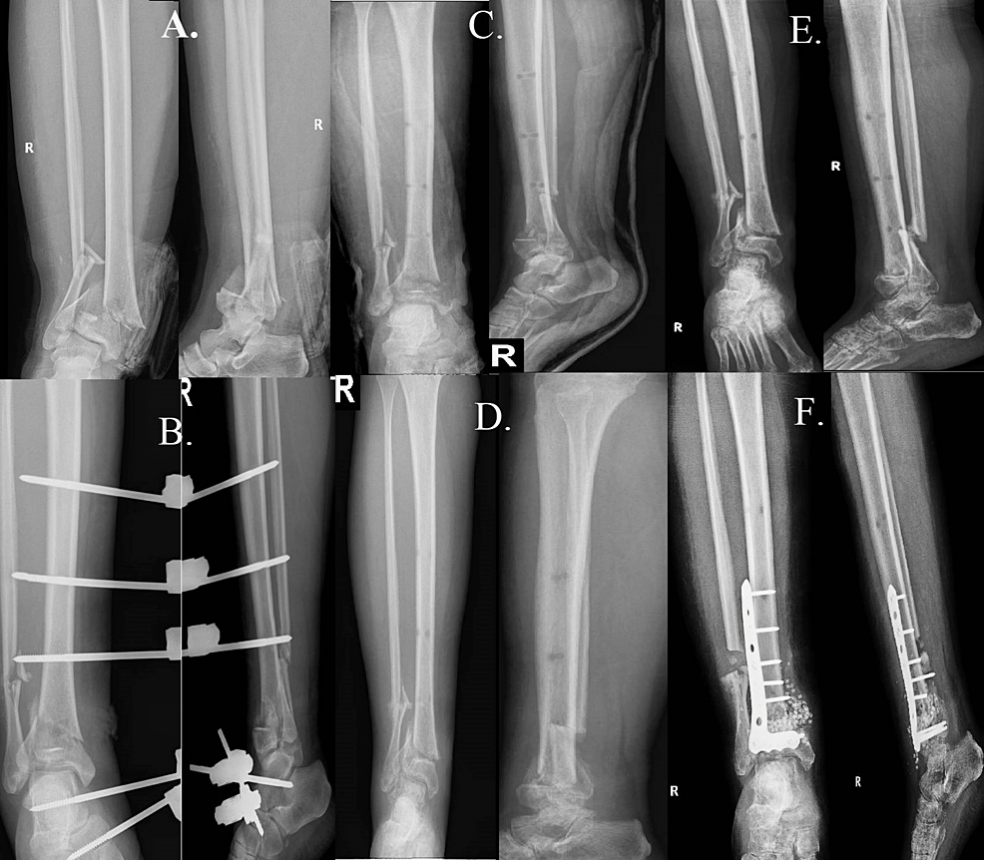
Case five A:  type IIIA Gustilo-Anderson (GA) fracture at the tibial pillar and fibula seen in a 36-year-old road accident victim. B: initial fracture stabilization with an external fixator. C: the patient refused the second stage of surgery, resulting in prolonged external fixator use. D: one month later, the patient's noncompliance with the treatment plan caused fracture site displacement. E: shows the patient's condition four months after the external fixator was removed, showing deviation and pain due to delayed surgery. Image F: surgical intervention was needed to osteotomy the fibula and re-fix the tibia malunited callus.

This case exemplifies the intricacy of a 36-year-old individual involved in a road accident, who exhibited a type IIIA Gustilo-Anderson (GA) fracture located at the tibial pillar and fibula (Figure 5A). The initial course of action involved the application of an external fixator, and the patient was duly informed about the need for a subsequent surgical intervention (Figure 5B). Nevertheless, the patient's noncompliance with the prescribed treatment led to a departure from the standard treatment protocol. The patient was advised to adhere to a treatment plan that involved maintaining the external fixator for a prolonged period of 45 days, followed by its removal and subsequent immobilization using a plaster cast because he refused the second stage of the surgical treatment. Additionally, the patient was strongly encouraged to attend weekly follow-up appointments for monitoring and evaluation. Nevertheless, the patient's failure to adhere to the prescribed treatment resulted in a subsequent visit after one month, during which a displacement was observed at the site of the fracture (Figure 5C). The patient was informed about the potential for an adverse outcome, yet once again declined to undergo surgical intervention. Four months following the removal of the external fixator, the patient presented with notable deviation and pain, indicating a more complex situation (Figures 5D, 5E). In the end, the patient provided consent for surgical intervention; however, the delay in obtaining consent further aggravated the issue. Surgical intervention involving osteotomy of the malunited callus located at the fibula, followed by osteotomy and subsequent re-fixation of the malunited callus at the tibia, was deemed necessary (Figure 5F). This case underscores the significant significance of patient adherence and the intricate equilibrium that healthcare providers must uphold in regard to both honoring patient autonomy and adhering to optimal medical protocols.

Case six

**Figure 6:**
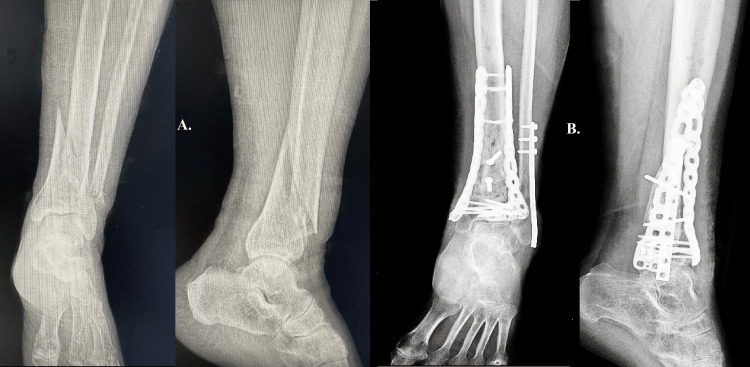
Case six A: a spiral fracture in the distal third of the tibia and a fibula fracture. B: the patient seeks urgent care for tendinitis after vigorous physical activity 15 months after surgery.

This case examines a 76-year-old individual with a documented record of alcohol consumption. The individual in question encountered a low-energy traumatic incident subsequent to a descent from an elevated stool, while concurrently exhibiting heightened blood alcohol levels upon their arrival at the emergency department. The occurrence of a spiral fracture in the distal third of the tibia, accompanied by a concurrent fracture of the fibula, was a direct result of the traumatic incident (Figure 6A). Considering the patient's advanced age, compromised bone quality, susceptibility to non-union, and potential for poor treatment adherence, along with the low-energy nature of the trauma and the relatively favorable condition of the soft tissues, it was concluded that the optimal approach would involve performing reduction and osteosynthesis. The surgical intervention involved the placement of two plates at the tibial location, along with the insertion of a single plate at the fibular site. Despite exhibiting a favorable postoperative course, the patient demonstrated non-adherence to the prescribed re-evaluation protocols. The data was collected approximately 15 months after the surgical procedure when the individual sought immediate medical care as a result of tendinitis following a period of intense physical exertion (Figure 6B). The provided text offers significant insights into the challenges and complexities involved in managing fractures in patients with unique risk factors and behavioral patterns. The aforementioned findings emphasize the importance of customizing treatment approaches to accommodate the unique requirements of each individual.

Case seven

**Figure 7:**
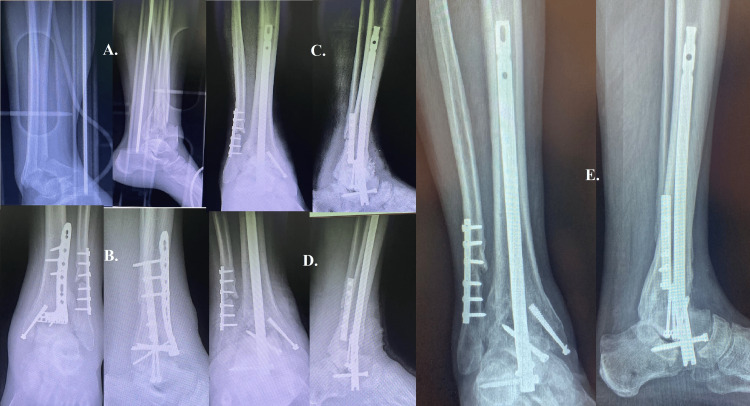
Case seven A: shows the comminuted fracture, which was hard to spot due to the patient's restlessness. B: shows fracture reduction and fixation three weeks after the fracture, with fibrosis preventing precise anatomical alignment. C: shows the postoperative radiograph two years later with a centromedullary rod with a bone substitute used to treat tibia-calcaneal-talar arthrosis. D: shows arthrodesis one month after intervention. E: successful arthrodesis one year after surgery, with the patient walking almost normally.

The case under consideration pertains to a notably intense event, wherein a middle-aged individual of 56 years of age, subsequent to a deliberate act of self-harm involving jumping out of a window from the eighth floor, was admitted to the emergency department of the university hospital (Figure 7A). The patient presented with a multifaceted injury pattern, including hemopneumothorax, costal flap, fractures of four ribs on the left side, multiple fractures at the thoracic and lumbar vertebral bodies, moderate craniocerebral trauma, spleen rupture, and a comminuted fracture of the tibial pillar. Considering the patient's physical condition and underlying psycho-emotional disorders, it was necessary to carefully deliberate on immediate treatment strategies. The fracture of the tibial pillar, specifically the comminuted type, required a careful and deliberate approach. In the given scenario, the utilization of external fixation for fracture stabilization, although commonly recommended, would have posed significant risks. The potential risk associated with the patient's physical and mental condition extends not only to the patient himself but also to the medical staff responsible for his care, as there is a possibility of self-inflicted or other-directed harm. Consequently, the medical team made the decision to employ immobilization through the utilization of a splint until the patient's condition was stabilized. The decision was motivated by the wider context of the individual's numerous traumatic experiences and the need for a comprehensive and coordinated approach to their healthcare. The presence of the patient's restlessness and concurrent pathologies posed challenges in obtaining a high-quality radiograph, thereby unveiling the comminuted nature of the fracture. The radiography demonstrates the utilization of fracture reduction techniques, immobilization methods, and the application of bone substitutes (Figure 7B). The completion of this stage was delayed by three weeks following the fracture due to the presence of associated pathologies. The development of fibrosis hindered the achievement of precise anatomical alignment. The development of tibia-calcaneal-talar arthrosis can be attributed to the patient's lack of adherence to medical recommendations and the intricate nature of the fracture. After a span of two years, a centromedullary rod accompanied by a bone substitute was employed for the purpose of rectifying the aforementioned condition (Figure 7C). The radiography provided pertains to the radiographic image taken post-surgery. One month following the implementation of the intervention (Figure 7D), the radiography demonstrates observable advancements in arthrodesis. The postoperative assessment, conducted one year following the surgical procedure, demonstrates a favorable outcome characterized by a successful arthrodesis (Figure 7E). Significantly, the patient exhibited nearly normal ambulatory capabilities. This particular case underscores the significance of patient compliance, prompt intervention, and adaptable surgical strategies in the context of severe trauma. The chosen course of action emphasizes the significance of personalized and comprehensive patient care, considering not only the physical traumas but also the underlying psychological state and the well-being of both the patient and the healthcare staff. The aforementioned case highlights the complex and multifaceted aspects of trauma care, particularly when combined with intricate psychological and emotional factors. It also emphasizes the importance of flexible and patient-focused approaches in the field of emergency medicine.

## Discussion

The management of fractures involving the tibial pillar presents a multifaceted and intricate challenge within the field of orthopedics. This article provides a comprehensive analysis of the different approaches used in the treatment of these fractures, considering factors such as the specific type of fracture, the state of the surrounding soft tissues, the level of patient adherence, and any concurrent medical conditions. Complete restoration of ankle function is typically not attained in cases of surgical pilon fractures. In the existing literature on postoperative ankle functions, the prevalence of favorable outcomes, categorized as good or excellent, exhibits considerable variability, yet generally remains suboptimal. In the study conducted by Jansen et al., a total of 35 pilon fractures were examined, and it was observed that the average American Orthopaedic Foot and Ankle Society (AOFAS) score was 65, indicating poor outcomes [10]. Similarly, Ketz et al. reported an average AOFAS score of 85.2 in nine patients who underwent posterior plaque application to the distal tibia, while 10 patients who underwent a standard anterior approach had an average score of 76.4, indicating moderate outcomes [11]. In their study, Zhao et al. [12] documented a success rate of 81% in the implementation of absorbable implants, categorizing the outcomes as good and excellent.

The feasibility and efficacy of a single-stage treatment approach utilizing open reduction and internal fixation (ORIF) were demonstrated in our study, particularly in cases characterized by high-quality soft tissues. The utilization of a 3.5 mm locked anteromedial pillar plate or a 3.5 mm locked distal tibia anatomical plate, in conjunction with cannulated screws, played a crucial role in attaining the desired alignment. A Kirschner pin is typically used at the level of the fibula in our clinic because it, along with the plate at the level of the tibia, offers adequate stability and significantly lowers the risk of skin necrosis, a problem described in the literature when we discuss two incisions at this level.

The intricacy of soft tissue damage is a noteworthy consideration, impacting both approaches to treatment and resulting outcomes. A severe soft tissue injury has been found to significantly increase the duration of hospitalization, with an average stay ranging from 4.4 to 11.8 days. Furthermore, the costs associated with such injuries are substantially higher, reaching up to ten times the expenses incurred by non-displaced fractures that involve minimal tissue damage. The aforementioned statement highlights the significance of soft tissue status (STS) in not only providing guidance for treatment but also serving as a predictive factor for outcomes subsequent to extremity fractures [13,14]. The impact of trauma energy on bone and the adjacent soft tissue is consistently observed, although the latter is frequently given limited attention. The present preliminary study investigates methods for quantitatively evaluating the effects of trauma on secondary traumatic stress, which involves observing heightened blood flow and tension in damaged tissues of closed fractures. The cascade resulting from trauma triggers a series of biological and biomechanical alterations, such as significant hemorrhage and edema, disruption of the microvascular system, and intensified inflammatory responses [15,16]. These modifications increase the likelihood of complications, infections, and hindered healing, leading to the adoption of staged management and treatment approaches guided by soft tissue. The utilization of optical sensors to assess heightened hemorrhage and blood flow, along with the implementation of tactile measures to quantify tissue tension, presents a standardized approach for evaluating STS. The incorporation of these treatment strategies into existing protocols represents a positive advancement in the field of closed ankle fracture management, indicating a move towards a more thorough and refined comprehension of the subject matter [13,17].

A minimally invasive approach was utilized in patients who had a high risk of complications during surgery due to the presence of associated pathologies. This resulted in a positive outcome while maintaining mechanical stability, thereby taking into account the patient's overall health condition. The study revealed that patient non-compliance had a significant impact, highlighting the challenges associated with it. Noncompliance with the prescribed treatment regimen resulted in the postponement of necessary medical intervention, heightened intricacy of the surgical operation, and posed a potential threat of an unfavorable final result. This statement emphasizes the significance of patient education and collaborative decision-making within the field of medicine [3, 7,18].

The tabulated data (Table 1) provides a comprehensive synthesis of multiple studies pertaining to complications associated with tibial pilon fractures and related fractures. The provided information encompasses various research articles and methodologies, providing a concise overview of significant complications such as superficial and deep infections, osteoarthritis, non-union, malunion, delayed union, amputations, wound problems, and other specific concerns. Additionally, it furnishes comprehensive information regarding the number of cases examined and the proportion or precise incidence of each complication. The treatment approach for open fractures, especially those classified as Gustilo-Anderson type II or higher, prioritized a two-stage methodology. The utilization of an external fixator for initial stabilization, followed by open reduction and internal fixation (ORIF), effectively reduced the likelihood of septic complications. The implementation of this approach involved diligent observation of soft tissue edema and the maintenance of adequate blood flow, which were crucial factors. When citing the table, it is crucial to recognize the limitations inherent in the data. Although our objective was to provide a comprehensive overview, it is apparent that not all studies included information on every metric. This underscores the need for increased implementation of standardized reporting in future studies pertaining to the subject matter.

**Table 1:** Complications associated with tibial pilon fractures By integrating data from various studies, this table provides a comprehensive analysis of the complications associated with tibial pilon fractures. Encompassing a range of medical conditions spanning from infections to amputations, this study underscores the significance of a two-stage therapeutic approach in managing open fractures. Specifically, it underscores the criticality of initial stabilization followed by subsequent open reduction in achieving optimal patient outcomes. This comprehensive compilation of methodologies offers a distinctive perspective for comparing the intricacies of various research approaches, emphasizing the objective of reducing septic complications. The abbreviation "N.R." is used to denote instances where data has not been reported or explicitly mentioned in the study under consideration.

Complication	Carbonell-Escobar et al. [22]	Liu et al. [2]	Van den Berg et al. [23]	Danoff et al. [24]	Sajjadi et al. [25]	Janssen et al. [26]	Daniels et al. [27]	Chaudhry et al. [28]	Harris et al. [29]	Gulbrandsen TR et al. [30]
Open fractures (%)	23.91	N.R	15.3	N.R	N.R	63	N.R	39.1	27	N.R
Mean follow-up (months)	39	39.6	49.9	N.R	N.R	N.R	N.R	59.82	26	N.R
Superficial Infection (%)	7.6	4.17	9.3	N.R	9.5	6	6.8	N.R	2.5	45
Deep Infection (%)	8.7	N.R	3.4	14.3	2	6	12.4	N.R	1.3	45
Osteomyelitis (%)	N.R	N.R	N.R	N.R	0	N.R	N.R	N.R	N.R	N.R
Non-union (%)	10.9	N.R	4.2	7.1	4.8	12	1.4	2.9	2.5	30
Malunion (%)	N.R	4.17	N.R	N.R	9.5	12	1.2	2.9	5.1	N.R
Amputation (%)	N.R	N.R	N.R	N.R	3	5.3	1.2	0	N.R	12

Although various factors such as age, gender, and social status have been observed to influence functional outcomes, the primary predictors for physical and functional outcomes appear to be the severity of the fracture and soft tissue damage. Additionally, the quality of reduction, congruence of the articular surface, and axial alignment of the tibiotalar joint have been identified as significant factors. This section discusses the limitations of the study and suggests potential areas for future research [19-21]. The study's exclusion criteria, which encompass factors such as pediatric fractures, and stress fractures, among others, serve to provide specific and targeted insights. However, it is important to acknowledge that these criteria may also restrict the generalizability of the findings. Subsequent investigations may delve into the aforementioned categories that were omitted, alongside conducting extended follow-up studies to evaluate functional outcomes.

## Conclusions

The optimal treatment for ankle fractures depends on assessing bone and soft tissue damage. A thorough analysis of closed pillar fractures and Gustillo-Anderson-classified open injuries shows the importance of tailored and sequential methods. A single-step reduction-fixation strategy works when soft tissue quality is good. However, open fractures require careful two-step surgery. The novel combination of optical and tactile soft tissue assessment methods adds complexity to our understanding of trauma's effects.

Comprehensive insights from this discourse emphasize the importance of personalized therapeutic strategies. These strategies must take into account the fracture's severity, surrounding soft tissues, the patient's health, and their medical compliance. Innovation in measuring and evaluating ankle fracture management surgical techniques and protocols is crucial to achieving optimal outcomes and minimizing complications in this complex field.
